# Short Telomeres in ESCs Lead to Unstable Differentiation

**DOI:** 10.1016/j.stem.2013.01.018

**Published:** 2013-04-04

**Authors:** Fabio Pucci, Laura Gardano, Lea Harrington

**Affiliations:** 1 Wellcome Trust Centre for Cell Biology and Institute of Cell Biology, School of Biological Sciences, Michael Swann Building, The University of Edinburgh, Edinburgh EH9 3JR, Scotland, UK; 2 Institut de Recherche en Immunologie et en Cancérologie, Université de Montréal, 2950 Chemin de Polytechnique, Pavillon Marcelle-Coutu, Montréal, Québec H3T 1J4, Canada

## Abstract

Functional telomeres are critical for stem cell proliferation; however, whether they are equally important for the stability of stem cell differentiation is not known. We found that mouse embryonic stem cells (ESCs) with critically short telomeres (*Tert*^*−/−*^ ESCs) initiated normal differentiation after leukemia inhibitory factor (LIF) withdrawal but, unlike control ESCs, failed to maintain stable differentiation when LIF was reintroduced to the growth medium. *Tert*^*−/−*^ ESCs expressed higher levels of Nanog and, overall, had decreased genomic CpG methylation levels, which included the promoters of *Oct4* and *Nanog*. This unstable differentiation phenotype could be rescued by telomere elongation via reintroduction of *Tert*, via suppression of Nanog by small hairpin RNA (shRNA) knockdown, or via enforced expression of the de novo DNA methyltransferase 3b. These results demonstrate an unexpected role of functional telomeres in the genome-wide epigenetic regulation of cell differentiation and suggest a potentially important role of telomere instability in cell fate during development or disease.

## Graphical abstract

**Graphical Abstract:**

**Figure:**
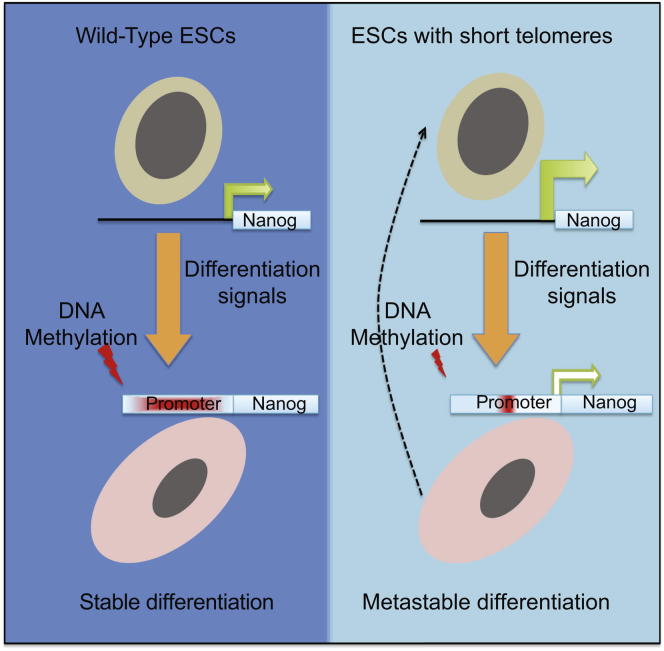

**Highlights:**

► ESCs with short telomeres do not stably differentiate and can resume proliferation ► Critically short telomeres impair Nanog regulation during ESC differentiation ► Short telomeres elicit genome-wide DNA hypomethylation and altered H3K27me3 levels ► Impaired DNA methylation and differentiation are rescued by Dnmt3b or telomere elongation

## Teaser

Functional telomeres not only are important for stem cell proliferation but, in ESCs, are required to maintain stable differentiation in part by regulating Nanog expression

## Introduction

Murine embryonic stem cells (ESCs) are self-renewing, pluripotent cells able to differentiate into cells of all three germ layers. Pluripotency and self-renewal are maintained primarily by the core transcriptional factors Nanog, Oct4, and Sox2 (Heng et al., 2010) but require both the cooperation of other factors and coregulators (Li, 2010) and an efficient telomere maintenance mechanism (Huang et al., 2011). In mammals, telomere maintenance is achieved via a telomerase reverse transcriptase (*Tert*) and an integral RNA component (*Terc*) that synthesize new telomeric DNA during cell proliferation. An appropriate telomere maintenance system is important for ESC replicative potential (Agarwal et al., 2010; Batista et al., 2011; Marion et al., 2009). During the reprogramming of differentiated cells into stem cells, an increase in telomerase activity leads to telomere elongation and the acquisition of epigenetic marks characteristic of longer telomeres (Marion et al., 2009). Notably, the teratoma-forming ability of ESCs derived from late generation (G3–G4) *Terc*^*−/−*^ mice with critically short telomeres is greatly reduced (Huang et al., 2011).

## Results

### Critically Short Telomeres in ESCs Lead to Elevated Basal Levels of Nanog

**Figure 1:**
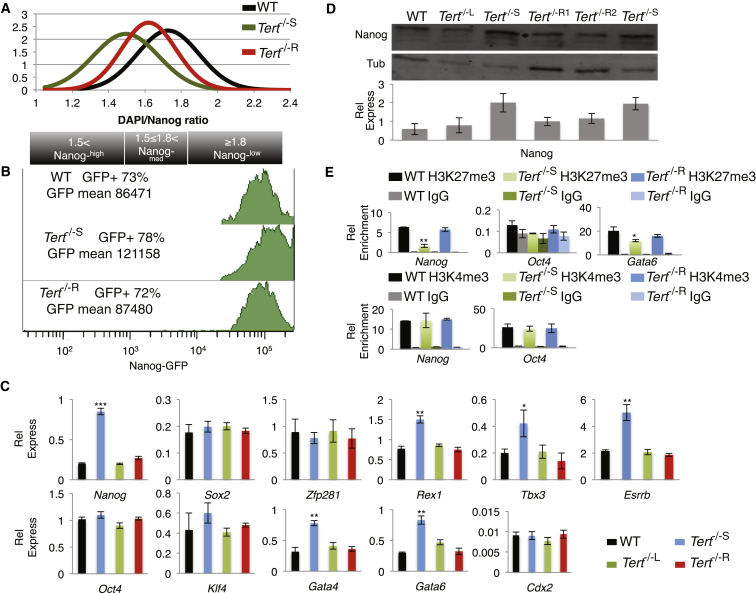
Analysis of Pluripotency Factors in WT and *Tert*^−/−^ ESCs (A) Quantification of Nanog levels normalized over DAPI (see Figure S1G for corresponding immunofluorescence images). Note a significant shift (p < 0.0001) from Nanog-low (DAPI to Nanog-488 ≥ 1.8) to Nanog-high (DAPI to Nanog-488 < 1.5) cells in *Tert*^−/−S^ in comparison to WT and *Tert*^−/−R^ ESCs (n ≥ 100 per cell population). (B) FACS analysis of the Nanog expression profile in the same genotypes as in (A). Note the rightward shift and increase in average Nanog signal intensity in *Tert*^−/−S^ ESCs. (C) Relative gene expression analyzed by qRT-PCR, normalized to *GAPDH* (n = 4). Data are represented as mean ± SD. (D) (Top) Nanog protein expression with LI-COR quantification below (n = 3). Data are represented as mean ± SD; L, long telomeres (passage 30); S, short telomeres (passage 70); R, *Tert* rescue (70 passages, followed by clonal selection and an additional 4 passages after *Tert* reintroduction). The superscripts 1 and 2 indicate two independently generated *Tert*^−/−R^ colonies. (E) ChIP analysis using an antibody to H3K27me3 and H3K4me3. Relative enrichment was quantified with the use of region-specific qPCR primers for *Nanog*, *Oct4*, and *Gata6* promoters. Generic IgG was used as a control (n = 3). Data are represented as mean ± SD. ^∗^, p < 0.05; ^∗∗^, p < 0.01; ^∗∗∗^, p < 0.0001. See also Figure S1 and Table S1.

We sought to address the impact of telomere dysfunction not only upon the capacity for cell differentiation but also upon the maintenance of a differentiated state. Late-passage *Tert*^−/−^ ESCs (*Tert*^−/−S^) (Liu et al., 2000) that possessed shorter telomeres and a significant accumulation of telomere signal-free ends relative to wild-type (WT) ESCs or *Tert*^−/−^ cells at earlier passages (*Tert*^−/−L^) (Figures S1A–S1C available online; p < 0.0001; Fisher’s exact test) were nonetheless proliferation-competent and did not exhibit an altered doubling time, cell morphology, or cell-cycle distribution (Figures S1D and S1E; data not shown). However, *Nanog* messenger RNA (mRNA) and protein levels were significantly elevated (Figures 1A–1C and S1F–S1G). No difference was observed in *Oct4*, *Sox2*, and *Klf4* expression (Figures 1C and S1F). To test whether the difference in Nanog expression was related to telomere dysfunction, we reintroduced WT *Tert* into late-passage *Tert*^−/−^ ESCs (*Tert*^−/−R^), and, after the propagation of clonal lines expressing *Tert*, we observed the reparation of telomere signal-free ends and a restoration of Nanog levels closer to the levels observed in WT ESCs and *Tert*^−/−^ ESCs at early passage (Figures 1A–1D, S1A–S1C, S1F, and S1G). Transient expression of *Tert* for 72 hr, a period of time insufficient to permit telomere extension, failed to restore Nanog to levels comparable to WT ESCs (data not shown). These data suggest that the dysregulation of Nanog in *Tert*^−/−S^ ESCs is a consequence of critically short telomeres.

ESCs that express high levels of Nanog tend to self-renew, whereas cells that express low levels of this factor tend to differentiate (Chambers et al., 2007; Savarese et al., 2009; Singh et al., 2007). Immunofluorescence analysis of *Tert*^−/−S^ ESCs cultured on gelatin in leukemia inhibitory factor (LIF)-containing media revealed a significant increase in the percentage of Nanog^high^ cells in comparison to WT and *Tert*^−/−R^ ESCs (Figures 1A and S1G) (Savarese et al., 2009). We confirmed elevated Nanog expression in *Tert*^−/−S^ ESCs via fluorescence-activated cell sorting (FACS) analysis (Figure 1B). We also measured the expression of other factors involved in the pluripotency regulatory network (*Rex1*, *Esrrb*, and *Tbx3*) (Festuccia et al., 2012; Ivanova et al., 2006; Shi et al., 2006), including pluripotency factors that negatively regulate Nanog expression (*Zfp281*) (Fidalgo et al., 2011) and lineage differentiation markers (*Cdx2*) and the endoderm markers (*Gata6* and *Gata4*) that are negatively regulated by Nanog (Singh et al., 2007). As anticipated, *Rex1*, *Esrrb*, and *Tbx3* mRNA levels were increased in *Tert*^−/−S^ ESCs, whereas *Zpf281* and *Cdx2* levels were unaffected (Figure 1C). However, *Gata6* and *Gata4* were also increased (Figure 1C). Consistent with these observations, chromatin immunoprecipitation (ChIP) analysis revealed lower levels of Nanog occupancy on the *Gata6* promoter (Figure S1I). Nevertheless, the recruitment of Nanog to its own promoter, which represses its own expression (Fidalgo et al., 2011), increased in *Tert*^−/−S^ ESCs (Figure S1I). Thus, the increased expression of *Nanog* is not a consequence of the impaired occupancy of Nanog on its own promoter.

### Perturbations in H3K27me3 Are Associated with Critically Short Telomeres

Telomere attrition is associated with the loss of certain heterochromatin markers and DNA hypomethylation at telomeric and subtelomeric regions (Benetti et al., 2007). We postulated that the increase in *Nanog* expression could be linked to a general dysregulation of epigenetic repression, given that low levels of trimethylation on histone H3 lysine 27 (H3K27me3) promote *Nanog* and *Gata6* expression (Lu et al., 2011; Shen et al., 2008; Villasante et al., 2011). H3K27me3 was reduced at *Nanog* and *Gata6* promoters in *Tert*^−/−S^ ESCs, whereas H3K4me3 levels at the *Nanog* promoter were unaffected (Figure 1E). H3K27me3 and H3K4me3 enrichment on the *Oct4* promoter was unaffected (Figure 1E). These perturbations, including a slightly increased level of global H3K27me3 in *Tert*^*−/−*S^ ESCs, were restored upon telomere elongation (Figures 1E and S1H). These changes were not accompanied by a significant alteration in the three-dimensional localization of telomere DNA or chromatin in interphase nuclei (Figure S1J). Thus, the altered expression of *Nanog* and *Gata6* reflects changes in heterochromatin at their respective promoters independent of Nanog occupancy. Moreover, these results demonstrate that critically short telomeres also affect chromatin organization at loci distal to telomeres.

### Critically Short Telomeres Perturb the Ability of ESCs to Remain Stably Differentiated

**Figure 2:**
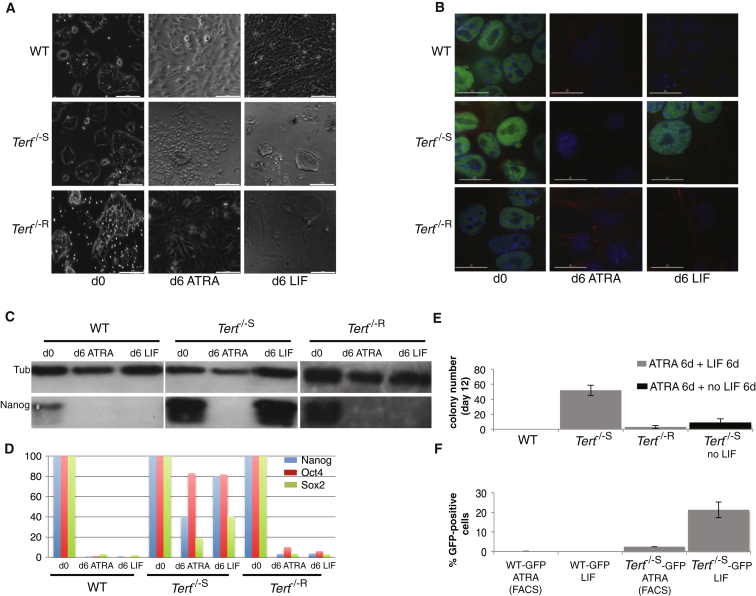
Differentiation Analysis of *Tert*^−/−^ ESCs (A) Bright field images of ESC populations at day 0 and day 6 in media containing 5 μM all-*trans* retinoic acid (ATRA) and, after ATRA removal, an additional 6 days in LIF-containing media. The micrograph bar indicates 200 μm for bright field images and15 μm for immunofluorescence images. (B) Nanog immunofluorescence analysis (green, Nanog; red, Actin). (C) Nanog protein detection by western blot. Tub, β-tubulin (n = 3). (D) qRT-PCR analysis of pluripotency genes after ATRA-induced differentiation. Gene expression at day 0 was arbitrarily set as 100, and the expression through the time course was normalized to mRNA levels at day 0. Values were expressed as a ratio to *GAPDH*. (E) Single-colony formation assay after ATRA treatment (6 days) is shown, and, where indicated, the readdition of LIF-containing media (6 days) (n = 3) is shown. The difference in the incidence of colony formation between *Tert*^−/−S^ (after LIF readdition) and all the other genotypes (or *Tert*^−/−S^ without LIF) was statistically significant (p < 0.0001; ANOVA and related Dunnett’s test comparing every group with *Tert*^−/−S^ values). The y axis indicates colony number. Data are represented as mean ± SD. (F) *Oct4*-promoter-driven GFP expression analysis of WT and *Tert*^*−/−S*^ ESCs post-ATRA treatment and after cell sorting for GFP-negative cells. The y axis indicates the percentage of GFP-positive WT, or *Tert*^−*/*−^ cells after 12 days of ATRA treatment and FACS sorting (FACS; columns 1 and 3) and after the readdition of LIF-containing media to GFP-negative sorted cells (columns 2 and 4). The difference in the incidence of GFP-positive cells between *Tert*^−/−S^ and WT cells was statistically significant (p < 0.00001; Welch’s unpaired t test). Data are represented as mean ± SD. See also Figure S2.

The impact of Nanog misregulation upon differentiation was tested by treating ESCs with 5 μM all-*trans* retinoic acid (ATRA), which was followed by the removal of ATRA and the readdition of LIF-containing media (Figure 2). Although longer ATRA treatment times were required to achieve suppression of *Oct4*, *Nanog*, and *Sox*2 mRNA and protein to levels comparable to WT or *Tert*^*−/−*^ ESCs with longer telomeres (Figures 2A−2D and S2), *Tert*^−/−S^ ESCs nevertheless exhibited a low proliferative capacity after ATRA treatment, which was consistent with a differentiated state (Figure 2E). However, after the readdition of LIF-containing media, *Tert*^*−/−*S^ ESCs failed to maintain repression of Nanog and exhibited robust colony formation only 6 days after the readdition of LIF-containing media (Figures 2 and S2). As an independent marker of differentiation, WT and *Tert*^*−/−*S^ cells were transduced with an *Oct4* promoter-driven green fluorescent protein (GFP) construct, treated with ATRA for 12 days, and then sorted to allow the selection of the GFP-negative population by FACS. Sorted GFP-negative cells were plated in the presence of LIF-containing media for 10 days, followed by an assessment of the percentage of GFP-positive cells. *Tert*^*−/−*S^ cells exhibited a high percentage of GFP-positive cells after the readdition of LIF-containing media (Figure 2F). These results demonstrate that ESCs with telomere dysfunction were able to execute only an incomplete, transitory repression of pluripotency genes in response to differentiation cues.

### ESCs with Short Telomeres Exhibit DNA Hypomethylation

**Figure 3:**
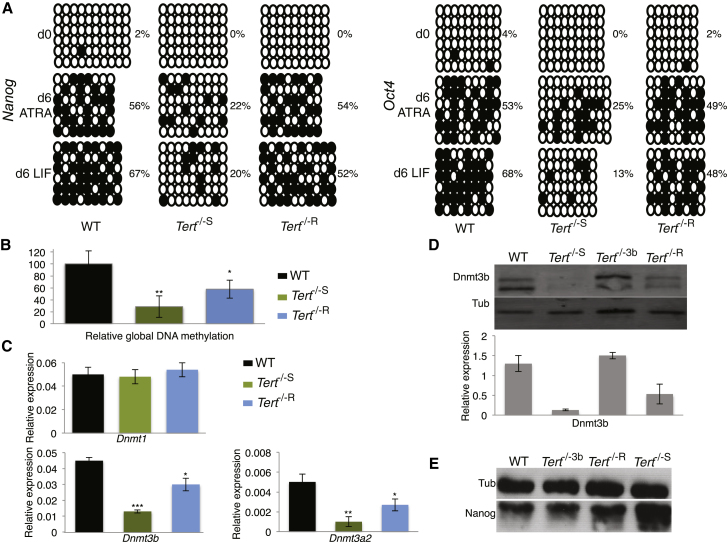
Expression of DNA Methyltransferases in ESCs Lacking *Tert* (A) CpG methylation analysis of the *Oct4* and *Nanog* promoters during ATRA treatment, followed by culture in LIF-containing media. Each column represents CpGs in a sequenced clone. Full dots symbolize methylated CpGs, and empty dots symbolize unmethylated CpGs. Percentage values indicate the proportion of methylated cytosine relative to total cytosine residues (n = 10). (B) Relative quantification of global DNA methylation (n = 3) is shown. Data are represented as mean ± SD. (C) Relative gene expression of *Dnmt1*, *Dnmt3b*, and *Dnmt3a2* analyzed by qRT-PCR. Values were normalized to *GAPDH* (n = 4). Data are represented as mean ± SD. (D) (Top) Dnmt3b protein detection by western blot and (bottom) after LI-COR quantification (n = 3). Data are represented as mean ± SD. (E) Nanog protein detection by western blot. Tub, β-tubulin (n = 5); R, *Tert* rescue; 3b, *Dnmt3b* rescue. Passage numbers are as in Figure 1. ^∗^, p < 0.05; ^∗∗^, p < 0.01; ^∗∗∗^, p < 0.0001. See also Figure S3.

Critically short telomeres are associated with DNA hypomethylation at subtelomeric DNA (Benetti et al., 2007). Given that we observed chromatin alterations at loci distal to telomeres, we tested whether *Tert*^−/−S^ ESCs also exhibited altered DNA methylation throughout the genome. Bisulphite-sequencing analysis of the *Nanog* and *Oct*4 promoters revealed a significant reduction in the acquisition of methylated cytosine in *Tert*^−/−S^ ESCs treated with ATRA relative to WT or *Tert*^−/−R^ ESCs (p ≤ 0.01 and p < 0.0001, respectively; Fisher’s exact test) (Figure 3A). Furthermore, *Tert*^−/−S^ ESCs failed to maintain even this level of cytosine methylation after the readdition of the LIF-containing media (p < 0.0001 and p = 0.03, respectively). At both promoters, this impairment was rescued in *Tert*^−/−R^ ESCs (p > 0.05; Figure 3A). Genome-wide methylation measured by an ELISA-based detection system against methylcytosine was also significantly reduced in *Tert*^−/−S^ ESCs (Figure 3B). Nonspecific epigenetic drift appeared improbable, given that WT and *Tert*^−/−R^ ESCs did not exhibit these changes after a similar propagation period. Although ESCs can tolerate DNA hypomethylation without impairment of cell proliferation (Tsumura et al., 2006), hypomethylation nonetheless impairs the capability of ESCs to achieve, and maintain a differentiated state (Feldman et al., 2006; Jackson et al., 2004; Sinkkonen et al., 2008). Thus, DNA hypomethylation in *Tert*^−/−S^ ESCs arose in response to critically short telomeres and impeded their stable differentiation.

### Restoration of Dnmt3b or Depletion of Nanog Rescue the Stable Differentiation of ESCs with Short Telomeres

**Figure 4:**
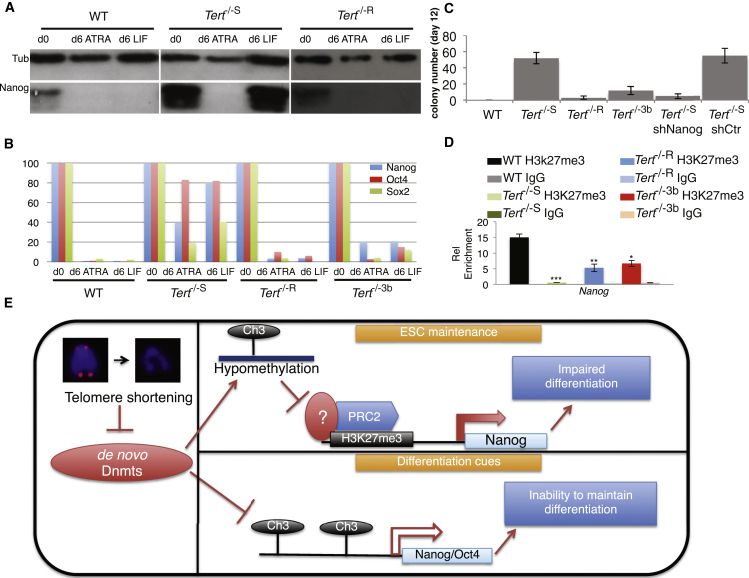
Differentiation Ability of *Tert*^−/−^ ESCs after Enforced Expression of Dnmt3b (A) Nanog protein detection by western blot. Tub, β-tubulin (n = 3). The first two panels on the left are reproduced from Figure 2C. (B) qRT-PCR analysis of pluripotency genes upon ATRA-induced differentiation. Gene expression at day 0 was arbitrarily set as 100 and the expression through the time course was normalized to mRNA levels at day 0. Values were expressed as a ratio to GAPDH. The first two genotypes were reproduced from Figure 2D. (C) Single-colony formation assay after the removal of ATRA and the readdition of LIF-containing media (n = 3). The difference in the incidence of colony formation between *Tert*^−/−S^ and all the other genotypes, apart from short hairpin control-transduced *Tert*^−/−s^ cells, was statistically significant (p < 0.0001; ANOVA and related Dunnett’s test comparing every group with *Tert*^−/−S^ values). The y axis indicates colony number. Data are represented as mean ± SD. (D) Chromatin immunoprecipitation analysis of H3K27me3 enrichment at the *Nanog* promoter, as described in Supplemental Experimental Procedures. Data are represented as mean ± SD (n = 3). ^∗^, p < 0.05; ^∗∗^, p < 0.01; ^∗∗∗^, p < 0.0001. (E) A schematic showing that telomere shortening impairs the expression of Dnmt3 isoforms, leading to genome-wide DNA hypomethylation, which, in turn, affects H3K27me3 enrichment on specific loci (e.g., *Nanog*), thus impairing the ability of ESCs to sustain pluripotency factor repression after differentiation and growth restimulation. See also Figure S4.

We tested whether the restoration of DNA methylation might restore the differentiation capability of *Tert*^−/−S^ ESCs. In mammals, genomic DNA methylation is principally regulated by three DNA methyltransferases (Dnmts): Dnmt1 (methylation maintenance) and the de novo methyltransferases Dnmt3a and Dnmt3b (Li et al., 1992; Okano et al., 1999). Although Dnmt1 expression was unaffected in *Tert*^−/−S^ ESCs, the expression of de novo methylases was reduced (Figure 3C). Enforced expression of Dnmt3b in *Tert*^−/−S^ ESCs restored repression of Nanog (Figures 3D, 3E, and S3(A) and restored the repression of *Nanog*, *Oct4*, and *Sox2* mRNA upon ATRA treatment (Figures 4A and 4B). Dnmt3b expression also led to a significant reduction in the colony formation of *Tert*^−/−S^ ESCs after the readdition of LIF-containing media (Figure 4C). The level of H3K27me3 at the *Nanog* promoter was also partially rescued in *Tert*^−/−S^ ESCs that expressed elevated Dnmt3b (Figure 4D). Consistent with a direct role of Nanog suppression in the maintenance of stable differentiation, Nanog depletion by small hairpin RNA (shRNA) was sufficient to overcome the inability of *Tert*^−/−S^ ESCs to remain differentiated (Figure 4C), and all genotypes transduced with *Nanog* shRNA exhibited a decrease in pluripotency gene expression (Figure S4). These results demonstrate that the mechanism of impaired ability to maintain stable differentiation in *Tert*^−/−S^ ESCs acts via the perturbation of de novo DNA methylation, which, in turn, influences chromatin organization and the ability to repress pluripotency factors such as *Nanog* under differentiation conditions.

## Discussion

Here, we report that critically short telomeres led to genome-wide DNA hypomethylation and that changes in H3K27 trimethylation occurred at loci distal to telomeres. The trimethylation of H3K27 is mediated by the polycomb repressive complex 2 (PRC2) and is associated with ESC identity (Shen et al., 2008). H3K27me3 is one of the principal histone repression markers, and its diminished enrichment on *Nanog* and *Gata6* promoters has been linked to the upregulation of these genes (Kim et al., 2008; Lu et al., 2011; Shen et al., 2008; Villasante et al., 2011). Although the global level of H3K27me3 increased in *Tert*^*−/−*S^ ESCs similar to recent studies that associate H3K27me3 enrichment with unmethylated CpG islands, its presence at *Nanog* and *Gata6* promoters was reduced (Lynch et al., 2012; Mendenhall et al., 2010). These data support the observation that DNA hypomethylation leads to overall increased levels of H3K27me3 in normally methylated regions but decreased levels of H3K27me3 in ordinarily unmethylated regions (Brinkman et al., 2012). Our data suggest a model whereby telomere-shortening-induced de novo Dnmt downregulation leads to DNA hypomethylation and altered H3K27me3 enrichment at promoters, which, in turn, affects the ability to repress pluripotency factors critical to stable differentiation in ESCs (Figure 4E).

The regulation of factors that affect pluripotency and differentiation are important not only to development but also to disease. For example, pluripotency factors such as Nanog tend to be highly expressed in undifferentiated tumors and in putative cancer stem cells (Tysnes, 2010). In addition, some cancer therapies employ differentiation-inducing agents such as retinoic acid in the treatment of acute promyelocytic leukemia (Petrie et al., 2009). Thus, it will be important to test whether critically short telomeres also influence cell fate in human cancer cells, particularly in the case of telomerase-inhibition strategies designed to instigate telomere instability.

## Experimental Procedures

### Cell Culture and Transfection

All experiments employed two separately generated ESC lines containing a disruption of endogenous *Tert*, as previously described (Liu et al., 2000). ESC lines were cultured on gelatin-covered dishes and maintained in Glasgow’s Modified Eagle’s Medium (GMEM; GIBCO) supplemented with 15% v/v fetal bovine serum (FBS), 0.055 mM β-mercaptoethanol (Sigma-Aldrich), 2 mM L-glutamine, 0.1 mM GMEM nonessential amino acids, 5,000 units/ml penicillin and streptomycin, 1,000 units/ml of recombinant LIF (Chemicon), and 1 μg/ml doxycycline and maintained at 37°C with 5% v/v CO_2_. To restore *Tert* expression to *Tert*^−/−S^ ESCs cells at passage, we cotransfected 70, ESCs with pTRE-Bi-*Tert*-IRES-EGFP-Hygro (or a similar vector lacking *Tert*) and CAG-rtTA advanced (pTET-ON advanced vector; Clontech). For constitutive expression of *Tert*, *Tert*^−/−S^ ESCs were transfected with CAG-*mTert*-IRES-Puro or CAG-IRES-Puro. For expression of Dnmt3b, *Tert*^−/−S^ ESCs were transfected with CAG-*Dnmt3b*-IRES-Puro or CAG-IRES-Puro. All transfections employed Fugene 6 (Roche) in a 3:1 ratio to DNA according to the manufacturer’s instructions. For *Tert* rescue or *Dnmt3b* reintroduction, cells were propagated for four passages under selection with hygromycin (500 μg/ml) or puromycin (5 μg/ml), and individual colonies were isolated. For *Nanog* shRNA transduction, cells were infected with commercially available lentiviral particles (Santa Cruz Biotechnology) and selected with puromycin (5 μg/ml). Cell transduction with *Oct4*-promoter GFP was performed by infection with commercially available lentiviral particles (System Biosciences). All lentiviral infections were performed in the presence of Polybrene (5 μg/ml; Santa Cruz Biotechnology). All experiments were performed with more than one clonal isolate.

### Differentiation Assay

Cell populations of the indicated genotype (1 × 10^5^) were plated in non-gelatin-covered dishes in LIF-free media containing 5 μM ATRA (Sigma-Aldrich) for the indicated amount of time with ATRA-media replaced every 3 days. At the indicated time point, cells were replated in gelatin-covered dishes with LIF-containing media. For the single colony formation assay, a set of serial dilutions was performed, and the number of viable ES cell colonies was assessed with alkaline phosphatase (Millipore).

### Quantitative Fluorescence In Situ Hybridization

The quantitative fluorescence in situ hybridization (Q-FISH) protocol was carried out as described previously (Liu et al., 2000). Metaphase spreads were captured with the use of Metafer 4 software and analyzed with Isis software. Statistical analysis of telomere intensity distribution was performed with Welch’s unpaired t test. The incidence of telomere signal-free ends was defined as the number of chromosome ends possessing a telomere signal (in arbitrary units) between 0 and 600, and statistical significance was assessed with Fisher’s exact test (InStat 3, GraphPad).

### qRT-PCR

Total RNA was isolated from cells with the use of Triazol (Invitrogen) according to the manufacturer’s instructions. Reverse transcription was carried out with the use of 0.5 μg of template RNA, random hexamer primers, and smart MMLV reverse transcriptase (Clontech). Diluted complementary DNA (20×) was subjected to real-time PCR analysis using a SYBR Green Master Mix (Roche) on a LightCycler 480 system (Roche). Background values (no reverse transcriptase added) were subtracted and values were normalized to *glyceraldehyde 3-phosphate dehydrogenase* (*GAPDH*) (n > 3). The oligos employed are listed in Table S1. Statistical analysis was performed by ANOVA and a related Dunnett’s test comparing every group with WT values.

### ChIP Sequencing

ChIP experiments were performed as described in Bergmann et al., 2011, except phenol-chloroform was replaced with a Chelex, 100-based DNA isolation method described in Nelson et al., 2006. Recovered DNA was analyzed by qRT-PCR as described above. For each pair of primers, triplicate measurements were taken and normalized to input DNA and the amount of DNA recovered from the *GAPDH* promoter (n > 3). Antibodies employed were as follows: rabbit anti-Nanog (Bethyl Laboratories); mouse anti-H3K27me3 and anti-H3K4me3 (Abcam); and murine IgG (Sigma-Aldrich). Oligos employed are listed in Table S1. Statistical analysis was performed by ANOVA and a related Dunnett’s test comparing every group with WT values. In each experiment, the signal present after immunoprecipitation with IgG was defined as background and subtracted prior to normalization to input DNA and *GAPDH*.

### Methylation Assay

Relative genomic DNA methylation was assessed with the use of the ELISA-based Imprint Methylated DNA Quantification kit (Sigma-Aldrich) according to the manufacturer's instructions, with the use of 100 ng of genomic DNA per sample (n > 3).

### Bisulphite Sequencing Analysis

DNA bisulphite conversion was performed as described previously (Clouaire et al., 2010). After bisulphite conversion of unmethylated cytosines to uracil, samples were resuspended in 1 × Tris–EDTA for PCR amplification. PCR products were cloned into pcDNA3.1 Directional TOPO Expression (Invitrogen) vector and colony PCR was performed. Clones (at least ten per sample) of the correct molecular mass were sequenced, and results were analyzed with BiQ Analyzer (http://biq-analyzer.bioinf.mpi-inf.mpg.de). Primers employed are listed in Table S1. Statistical analysis of samples employed Fisher’s exact test (two-sided) using GraphPad InStat3 (www.graphpad.com).

## Supplemental Information

Document S1. Supplemental Experimental Procedures, Figures S1–S4, and Table S1
