# Supplementary material for: Short Telomeres in ESCs Lead to Unstable Differentiation
Source: Cell Stem Cell. 2013 Apr 4;12(4):479–86. doi: 10.1016/j.stem.2013.01.018 (PMC3629568; doi:10.1016/j.stem.2013.01.018)
Supplement: Document S1. Supplemental Experimental Procedures, Figures S1–S4, and Table S1 [file mmc1.pdf]

Cell Stem Cell, Volume *12*

Supplemental Information

Short Telomeres in ESCs Lead to Unstable Differentiation

Fabio Pucci, Laura Gardano, and Lea Harrington

Supplemental Figures S1–S4

Supplemental Table S1

Supplemental Experimental Procedures

Supplemental References

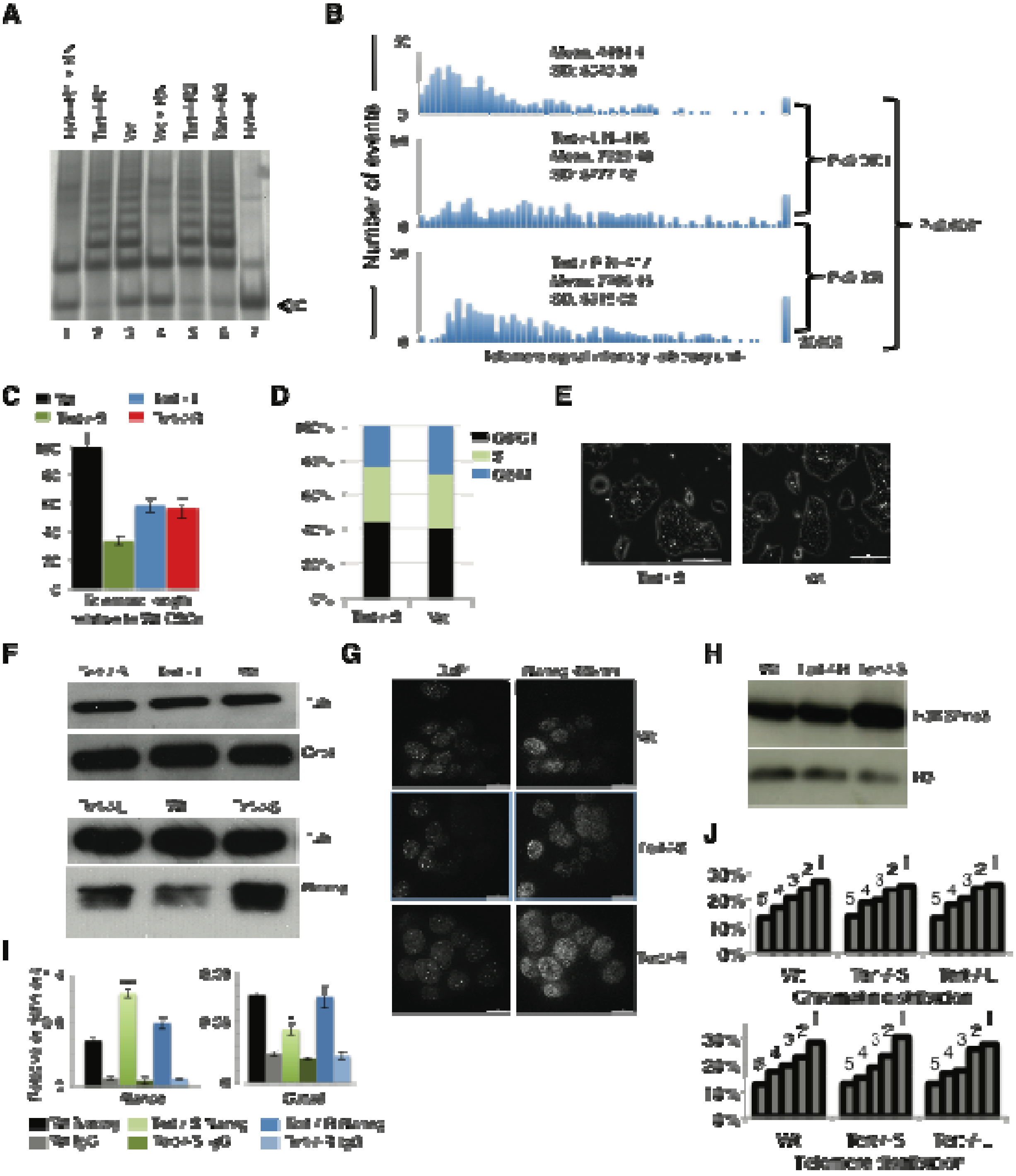
A
B
C
D
E
F
G
H
I
J
Number of events
G0/G1
S
G2/M
0%
20%
30%
20%
10%
0%
Chromatin distribution
Telomere distribution

Figure S1. Characterization of Telomerase Activity, Telomere Length, Cell-Cycle Profile, Morphology, and Pluripotency Gene Expression in *Tert*$^{-/-}$ ESCs, Related to Figure 1

A) Telomere repeat assay protocol (TRAP) performed on protein extracts (the equivalent of 5 x 10^4 cells) from Wt ESCs (lanes 1, 2), *Tert*$^{-/-R}$ ESCs (3 independent rescued clones; lanes 1, 2, 5, 6) untreated or after digestion with ribonuclease A (RN) and *Tert*$^{-/-S}$ ESCs (lane 7) (cells at between 67 and 74 passages); IC = internal PCR control.

B) Q-FISH analysis of indicated genotypes; statistical significance was analyzed by Welch's unpaired t-test; L = long telomeres (passage 30); S = short telomeres (passage 70); R = *Tert*$^{-/-S}$ cells after reintroduction of *Tert* (passage 74, including 4 passages under hygromycin selection). The difference in the incidence of signal-free ends relative to total ends between *Tert*$^{-/-S}$ (49/417) and *Tert*$^{-/-L}$ (14/416) or *Tert*$^{-/-R}$ (4/417) was statistically significant ($p<0.00001$ for each comparison, Fisher's exact test). N = number of chromosome ends; y-axis, number of events; x-axis, telomere signal intensity in arbitrary units.

C) Average of mean telomere signal intensity relative to Wt. Data are represented as mean ± SD (n=3); Number of chromosomes per sample ≥ 350

D) Cell cycle profile of Wt and *Tert*$^{-/-S}$ cells.

E) Bright field image of the same samples as in (D), Micrograph bars indicate 200μm.

F) Oct 4 and Nanog protein detection by western blot. β-Tubulin was used as an internal control (Tub). L = long telomeres (passage 70); S = short telomeres (passage 30); n=3 for Oct4 blot, n=10 for Nanog blot.

G) Immunofluorescence analysis of Nanog expression in ESCs. Micrograph bar indicates 15μm.

H) Detection of H3K27me3 and histone H3 (as a control) by western blot (n=3).

I) ChIP analysis of Nanog occupancy on *Nanog* and *Gata6* promoters (see Supplementary Experimental Procedures for details). A murine IgG antibody was used as a negative control. Data are represented as mean ± SD (n=3). * = $p<0.05$; *** = $p<0.0001$.

J) 3D-FISH analysis of chromatin (top) and telomere DNA distribution (bottom) in ESC nuclei (n=3). The nuclear area has been divided in 5 equal concentric zones, where 1 is the inner and 5 the most peripheral zone. Y-axis indicates the percentage of signal in each zone.

See also Supplemental Experimental Procedures.

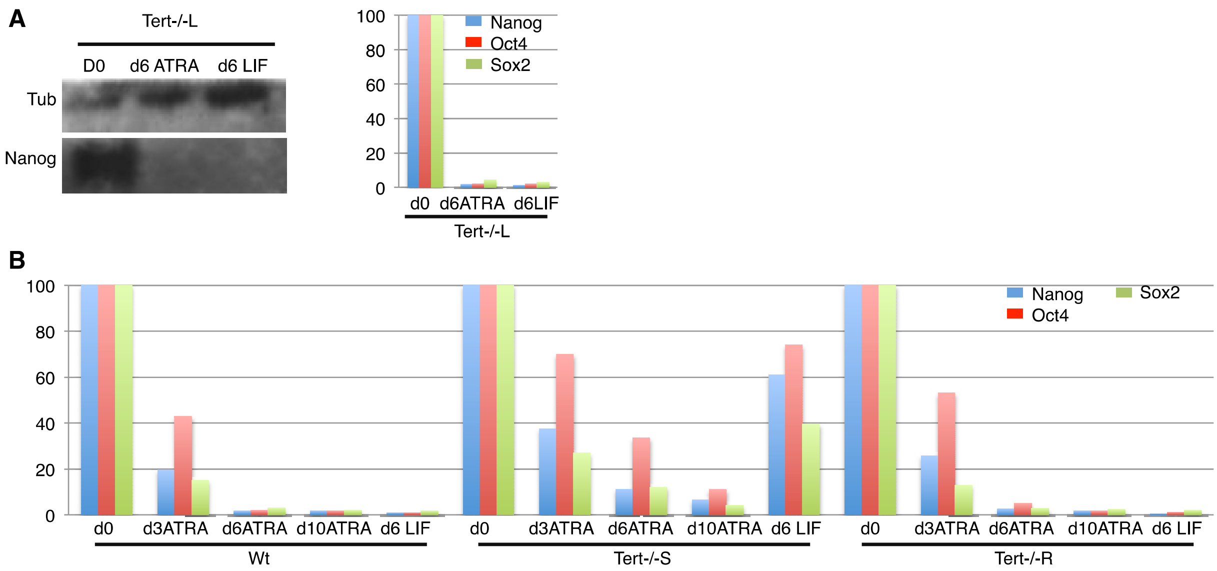

Figure S2. Analysis of *Nanog*, *Sox2*, and *Oct4* Expression in Response to All-*trans* Retinoic Acid and LIF, Related to Figure 2

A) Pluripotency factor expression in *Tert*$^{-/-}$ ESC with long telomeres (*Tert*$^{-/-L}$). (Left) Detection of Nanog and β-tubulin by western blot (n=3); (Right) QRT-PCR analysis of pluripotency genes after ATRA-induced differentiation and LIF re-addition. Gene expression at day 0 was arbitrarily set as 100 and the expression through the time course was normalized to mRNA levels at day 0. Sample nomenclature as indicated in Figure 1.

B) QRT-PCR analysis of pluripotency genes after ATRA-induced differentiation and LIF re-addition. Gene expression at day 0 was arbitrarily set as 100 and the expression through the time course was normalized to mRNA levels at day 0. Values were expressed as a ratio to *GAPDH*.

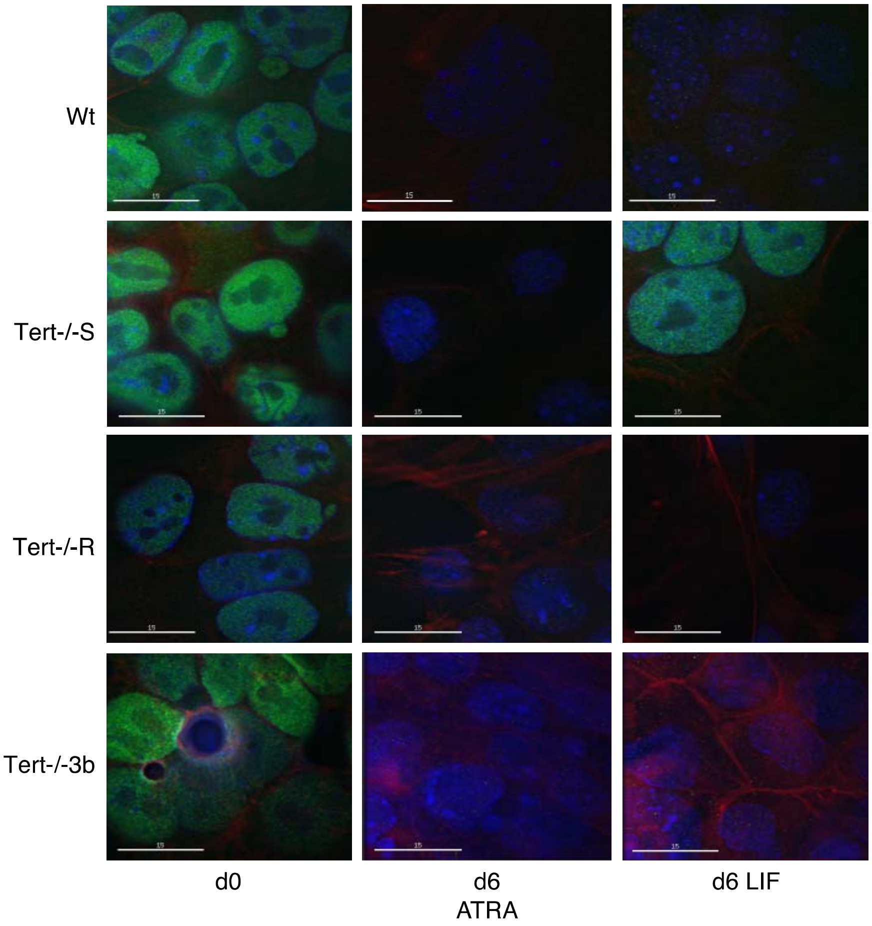

Figure S3. Nanog Immunofluorescence Analysis after Ectopic Expression of *Dnmt3b* in *Tert*$^{-/-}$ ESCs with Short Telomeres, Related to Figure 3

Cells were analyzed for Nanog via immunofluorescence at d0, day 0; d6 ATRA, 6 days of treatment with ATRA; d6 LIF, a further 6 days after removal of ATRA and re-addition of LIF-containing media. Note that the top nine panels are identical to Figure 2B, but are reproduced here because all samples were analyzed contemporaneously (n= 3). Micrograph bars indicate 15µm (n= 3).

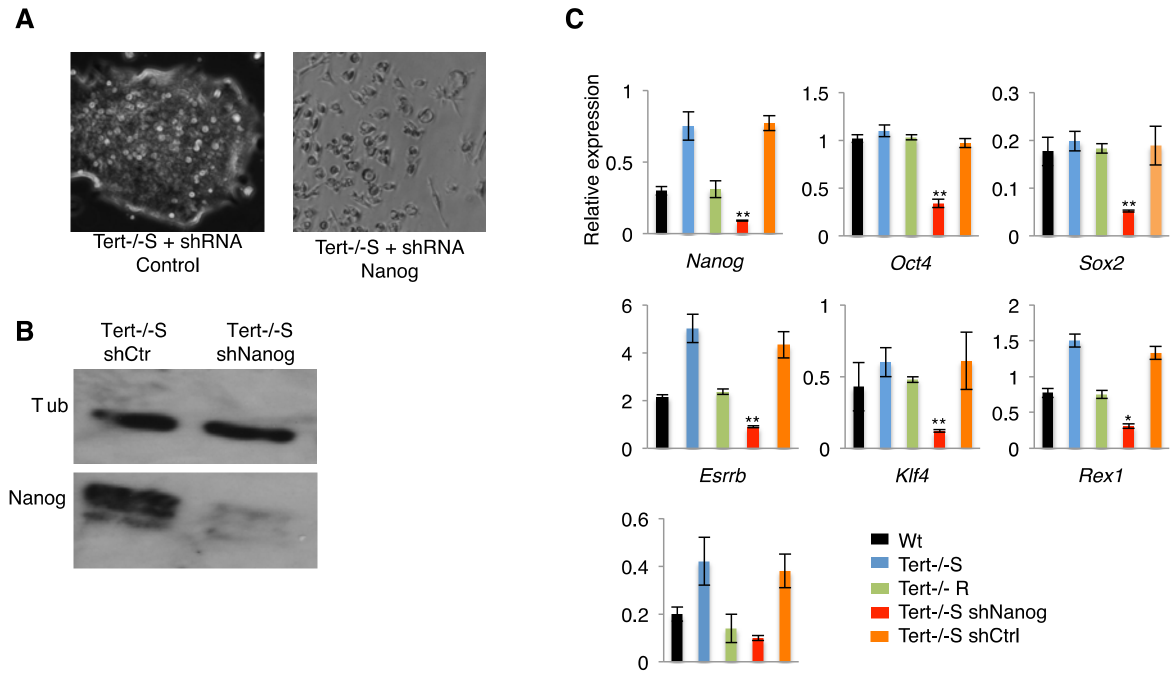

Figure S4. Knockdown of *Nanog* in *Tert*$^{-/-}$ ESCs, Related to Figure 4

A) Bright field image of shNanog and shControl transduced *Tert*$^{-/-}$ cells.

B) Nanog protein detection by western blot. Tub= β-tubulin (n=3).

C) Relative gene expression of shNanog and shControl transduced cells analyzed by QRT-PCR, normalized over GAPDH (n=3). Data are represented as mean ± SD. * = $p<0.05$; ** = $p<0.01$

Table S1. **List of Oligos Employed in This Study, Related to Figure 1**

**ChIP**	**Fw**	**Rv**
GAPDH[F]	AAGCTCATGAGGCACAGAATGGTC	TGGGTACATGGTGACTTTCCTAGGC
Gata6 [F]	TGACCCAGGAGGGGGCGAGT	CCGCCACCCAGGGCAGAAGA
Nanog [F]	ACTCCAAGGCTAGCGATTCA	AATAGGGAGGAGGGCGTCTA
Oct4[D]	CTGTAAGGACAGGCCGAGAG	CAGGAGGCCTTCATTTTCAA
**QRT-PCR**	**Fw**	**Rv**
Cdx2[I]	CCTGCGACAAGGGCTTGTTTAG	TCCCGACTTCCCTTCACCATAC
Dnmt1	TGGGCTGATGCAGGAGAAAAT	GCGCTTCATGGCATTCTCCTT
Dnmt3a2[S]	AGGGGCTGCACCTGGCCTT	TCCCCCACACCAGCTCTCC
Dnmt3b[S]	TGGGATCGAGGGCCTCAAAC	TTCCACAGGACAAACAGCGG
Esrrb[I]	CAGGCAAGGATGACAGACG	GAGACAGCACGAAGGACTGC
GAPDH[F]	CCATCACCATCTTCCAGG	CCTGCTTCACCACCTTCTTG
Gata4[F]	CTGTCATCTCACTATGGGCA	CCAAGTCCGAGCAGGAATTT
Gata6[F]	TTGCTCCGGTAACAGCAGTG	GTGGTCGCTTGTGTAGAAGGA
Klf4[T]	AGTGTGACAGGGCCTTTCCAGGT	AAGCTGACTTGCTGGGAACTTGACC
Nanog[F]	AGGGTCTGCTACTGAGATGCTCTG	CAACCACTGGTTTTTCTGCCACCG
Oct4[S]	GGCGTTCGCTTTGGAAAGGTGTTC	CTCGAACCACATCCTTCTCT
Rex-1[T]	CACCGACAACATGAATGAACAAAA A	CAATCTGTCTCCACCTTCAGCATT T
Sox2[T]	TAGAGCTAGACTCCGGGCGATGA	TTGCCTTAAACAAGACCACGAAA
Zfp281[F]	TGAGCCCAGGCACCCA	TGGAGAGGTGAA GACAAGCTGAC
**Bisulphite**	**Fw**	**Rv**
Nanog[T]	GATTTTGTAGGTGGGATTAATTGTGAATTT	ACCAAAAAAACCCACACTCATATCAATATA
Oct4[S]	AGGATTTTGAAGGTTGAAAATGAAGG	TCCCTCCCCAATCCCACCCTC

Superscripts indicate first author initial (Dahl et al., 2010; Fidalgo et al., 2011; Ivanova et al., 2006; Sinkkonen et al., 2008; Takahashi and Yamanaka, 2006). Fw, forward primer; Rv, reverse primer.

Supplemental Experimental Procedures

Telomerase Activity Assay

The telomere repeat amplification protocol, TRAP, was conducted with the TRAPeze Telomerase Detection Kit, Chemicon International, according to manufacturer's instructions.

Plasmid Construction

The plasmid pTRE-Bi-*Tert*-IRES-EGFP-Hygro was constructed by amplification of *Tert* cDNA by PCR and cloning it into pTRE-Tight-Bi (Clontech) following digestion with EcoRI and SalI. IRES-EGFP sequence was obtained from pCAGMKOSiE (from K.Kaji) and inserted into pTRE-Tight-Bi (following digestion with SalI and EcorV) using SalI and HpaI sites and then inserted into pTRE-Bi-*Tert* using NotI sites. Finally, the hygromycin-resistance gene was cloned by PCR into the XbaI restriction site of pTRE-Tight-Bi and pTRE-Bi-*Tert*-IRES-EGFP vectors to create pTRE-Bi-EGFP-Hygro and pTRE-Bi-*Tert*-IRES-EGFP-Hygro. The pCAG-rtTA-advanced vector was constructed by removal of the MKOS ORFs from CAGMKOSiE with EcoRI and BamHI and replacement with the advanced tetracycline reverse transactivator sequence (Clontech). The plasmid pCAG-*Dnmt3b*-IRES-puromycin vector was constructed by removal of the MKOS ORFs from CAGMKOSiE with EcoRI. *Dnmt3b* was subcloned from a *Dnmt3b* expression vector (Thermo scientific) and inserted pCAG-IRES-EGFP following digestion EcoRI and SalI. IRES-EGFP was replaced with IRES-Puro (from pIRESPuro2, Clontech) after digestion with PmlI and PvuII.

Fluorescence-Activated Cell Sorting (FACS)

Hoechst stain (5µg/ml) was added to the cell culture and incubated for 30 minutes. Cells were harvested and resuspended in 0.5 ml of 1X PBS and analyzed for cell cycle distribution using a Becton Dickinson Fluorescence Activated Cell Sorter. After gating on the appropriate channels, the percentage of cells in G1, S, or G2/M were calculated. For FACS analysis of Nanog expression, cells were fixed and stained as indicated (Festuccia and Chambers, 2011). Cell sorting after transduction with Oct4-GFP was carried out as described in Zheng and Hu, 2012.

Protein Extraction and Western Blot Analysis

Histones were acid-extracted as follows: Cells were harvested and washed twice with ice cold 1X PBS. Cells were resuspended (107 cells/ml) in TEB buffer (PBS 1X, 0.5% v/v Triton X-100, 2 mM PMSF, 0.02% v/v NaN_3) and left on ice for 10 minutes with gentle stirring to enhance lysis. Cells were spun at 800 x*g* for 10 minutes at 4°C, washed in TEB buffer, and pelleted as above. Cells were resuspended in 0.2 N HCl (4 x10^7 cells/ml) and incubated overnight at 4°C. Cells were pelleted as above, and the supernatant was recovered and stored at -80°C. Protein extracts were resolved on 15% w/v SDS-PAGE, transferred to nitrocellulose and blocked overnight with 3% w/v BSA in 1X PBS. Rabbit anti-histone H3 (Abcam) and mouse anti H3K27me3 (Abcam) were used as primary antibodies.

For non-histone protein extraction, cells were lysed for 30 minutes on ice in RIPA buffer (50 mM Tris-HCl pH 7.4, 1% v/v NP-40, 0.25% w/v Na-deoxycholate, 150 mM NaCl, 1 mM EDTA, 1 mM PMSF, 1 µg/ml each of aprotinin, leupeptin and pepstatin, 1 mM Na_3VO_4, 1 mM NaF). Cells were pelleted at 20,800 x*g* for 10 min at at 4°C. The supernatant was recovered and stored

at -80°C. Protein extracts were resolved on 10% w/v SDS PAGE, transferred to nitrocellulose and blocked overnight with 5% w/v non-fat dry milk in 1X PBS. Rabbit anti-Nanog (Bethyl labs), anti-Dnmt3b (Abcam) and Dnmt1 (Abcam), goat anti-Oct4 (Santa Cruz) and mouse anti-β-Tubulin (Sigma) were used as primary antibodies. Anti mouse and anti rabbit peroxidase-conjugated were used as secondary antibodies followed by detection with ECL Plus luminescent reagent (Amersham Biosciences) or with LI-COR in which instance the secondary antibodies employed were donkey anti-rabbit IRDye 800CW (green) and donkey anti-mouse IRDye680 (red) (Odyssey). All experiments were repeated at least three times.

Immunofluorescence

Cells were fixed in 4% v/v paraformaldehyde (PFA)/PBS according to manufacturer's instructions (Abcam). Rabbit anti-Nanog (Bethyl labs) and Alexa fluor® Goat anti-rabbit-488 were used as primary and secondary antibodies. Rhodamine-phalloidin (Sigma) was used to detect actin. DNA was stained with DAPI. ImageJ software was employed to define the relative fluorescence intensities of single cells (for channels 488), with DAPI fluorescence as internal control. Individual values were used for quantitative analysis of Nanog expression levels among genotypes as described (Savarese et al., 2009). Statistical analysis was performed using Welch's unpaired t-test.

3D Analysis of Cell Nuclei

Cells were fixed in in 4% v/v paraformaldehyde (PFA)/PBS for 15 min, then treated as described for Q-FISH analysis of telomere fluorescence. At least 25 interphase nuclei were analyzed for

Wt, *Tert*$^{-/-S}$ and *Tert*$^{-/-L}$. Images were acquired using a Nikon TE-2000 microscope equipped with a 1.45 numerical aperture 100× objective, PIFOC Z-axis focus drive (Physik Instruments), Sedat quad filter set, and CoolSnapHQ High Speed Monochrome charge-coupled device camera (Photometrics). Images were deconvolved from 0.2-µm sections using AutoquantX.

Deconvolved images were analyzed for chromosome and telomere distribution using a macro described in (Korfali et al., 2010).

Supplemental References

Dahl, J.A., Reiner, A.H., Klungland, A., Wakayama, T., and Collas, P. (2010). Histone H3 lysine 27 methylation asymmetry on developmentally-regulated promoters distinguish the first two lineages in mouse preimplantation embryos. PLoS One *5*, e9150.

Festuccia, N., and Chambers, I. (2011). Quantification of pluripotency transcription factor levels in embryonic stem cells by flow cytometry. Curr Protoc Stem Cell Biol *Chapter 1*, Unit 1B 9.

Korfali, N., Wilkie, G.S., Swanson, S.K., Srsen, V., Batrakou, D.G., Fairley, E.A., Malik, P., Zuleger, N., Goncharevich, A., de Las Heras, J., *et al.* (2010). The leukocyte nuclear envelope proteome varies with cell activation and contains novel transmembrane proteins that affect genome architecture. Mol Cell Proteomics *9*, 2571-2585.

Takahashi, K., and Yamanaka, S. (2006). Induction of pluripotent stem cells from mouse embryonic and adult fibroblast cultures by defined factors. Cell *126*, 663-676.

Zheng, X, and Hu, G. (2012). Oct4GiP reporter assay to study genes that regulate mouse embryonic stem cell maintenance and self-renewal. Journal of Visualized Experiments, May 30;(63). pii: 3987. doi: 10.3791/3987.
